# Influence of Abutment Configuration on the Fatigue Survival of Titanium Implant–Abutment Assemblies

**DOI:** 10.3390/ma19204374

**Published:** 2026-10-18

**Authors:** Ana Isabel Nicolas-Silvente, María Dolores Austro-Martinez, Loreto Monsalve-Guil, Eugenio Velasco-Ortega, Marta Carazo-Austro, Iván Ortiz-Garcia, Jesús Moreno-Muñoz, José Luis Rondón-Romero, Álvaro Jiménez-Guerra, Enrique Núñez-Márquez

**Affiliations:** 1 Restorative Dentistry, School of Dentistry, CEIR Campus Mare Nostrum, University of Murcia, 30008 Murcia, Spain; 2 Department of Dentistry, Universidad Cardenal Herrera-CEU Universities, 03202 Elche, Spain; maria.austromartinez@uchceu.es (M.D.A.-M.); carazomarta03@gmail.com (M.C.-A.); 3 Department of Stomatology, Faculty of Dentistry, University of Seville, 41009 Seville, Spain; lomonsalve@hotmail.es (L.M.-G.); ivanortizgarcia1000@hotmail.com (I.O.-G.); je5us@hotmail.com (J.M.-M.); jolurr001@hotmail.com (J.L.R.-R.); alopajanosas@hotmail.com (Á.J.-G.); enrique_aracena@hotmail.com (E.N.-M.)

**Keywords:** dental implant–abutment design, dental implants, materials testing, mechanical tests, proportional-hazards models, survival analysis

## Abstract

**Highlights:**

**What are the main findings?**

- Fatigue survival depended on cyclic load and abutment configuration.
- Each 10 N increase in Pmax was associated with a 2.52-fold higher fatigue hazard.
- MUSLA 04020 showed higher fatigue hazard than the other three configurations.

**What are the implications of the main findings?**

- Fatigue behaviour should be interpreted at the complete assembly level.
- Transmucosal height alone does not explain configuration-dependent fatigue survival.
- Censoring-aware survival analysis complements conventional implant fatigue descriptors.

**Abstract:**

Fatigue performance of implant-supported assemblies depends on both loading conditions and structural configuration. This study investigated whether abutment configuration influences fatigue survival in titanium implant–abutment assemblies after accounting for cyclic load magnitude. Thirty-six internal-connection assemblies representing four configurations (*n* = 9 each) were tested under cyclic loading according to ISO 14801. Cycles to fracture or test termination were analyzed using Cox proportional-hazards regression, with non-fractured specimens treated as right-censored and maximum cyclic load (Pmax) included as a covariate. Twenty-three specimens fractured and 13 were right-censored. Each 10 N increase in Pmax was associated with a 2.52-fold higher fatigue hazard (95% CI, 1.75–3.62; *p* < 0.001). Abutment configuration had a significant overall effect after load adjustment (*p* = 0.027). Compared with the reference configuration (MUSA 04010), MUSLA 04020 showed a higher fatigue hazard (HR = 10.89; 95% CI, 2.23–53.33; *p* = 0.003). After multiplicity correction, MUSLA 04020 also showed a higher fatigue hazard than MUSA 04050 and MUSLA 04060, whereas no significant differences were found among MUSA 04010, MUSA 04050, and MUSLA 04060. The principal association remained consistent in sensitivity and leave-one-out analyses. Fatigue survival was therefore configuration-dependent within the tested system and should not be attributed to transmucosal height alone. Specimen-level, censoring-aware survival modelling can complement conventional fatigue descriptors in standardized implant fatigue testing.

## 1. Introduction

Fatigue is an important determinant of the structural lifetime of implant-supported restorations because functional loading is repetitive rather than static [1]. Unlike monotonic failure, fatigue results from the accumulation of damage over repeated loading cycles, and specimens tested under nominally identical conditions may fail after substantially different numbers of cycles [1].

Fracture load alone therefore provides an incomplete description of mechanical durability. Fatigue behaviour also depends on the progression of damage during cyclic loading and on the number of cycles sustained before failure.

ISO 14801 establishes a standardized configuration for dynamic fatigue testing of endosseous dental implant assemblies and permits controlled mechanical comparisons between implant systems [2]. The test is not intended to reproduce physiological mastication or to predict clinical service life; its purpose is to provide reproducible laboratory conditions for comparative evaluation [2].

Within this framework, fatigue testing is widely used to assess the mechanical reliability of implant-supported restorations and to examine the effect of structural design on cyclic behaviour. The implant fixture, abutment, and prosthetic screw form a mechanically coupled assembly, so the measured response reflects their combined behaviour rather than that of an individual component [3].

Material properties are consequently only one component of the mechanical response. Assembly geometry and the transfer of cyclic forces across the implant–abutment interface also influence fatigue behaviour.

Previous experimental studies have examined internal-connection design, implant geometry, abutment configuration, restorative geometry, implant diameter, and effective structural cross-section as potential determinants of fatigue performance. Their effects are not uniform across implant systems, and both the magnitude and direction of the observed differences may depend on the specific design under investigation [4,5,6,7,8,9].

Macroscopic fracture pattern is likewise not sufficient to characterize fatigue behaviour. Configurations with similar terminal fracture locations may show different fatigue limits or load–life relationships [4,10]. Abutment geometry, including transmucosal height, has therefore received increasing attention as a potential contributor to fatigue performance in specific implant systems [7,8].

Fatigue limits, fracture loads, and load–life (S–N/F–N) relationships remain the conventional descriptors of cyclic performance [10,11,12]. These measures are essential for engineering comparisons, but they summarize a time-dependent process in a limited number of parameters.

A specimen that reaches the end of a test without fracture still provides information about its fatigue life, although its eventual failure time is unknown. Likewise, specimens tested at different cyclic load magnitudes experience different mechanical exposures that are not fully represented by group-level summaries [1,11].

Specimen-level survival analysis permits cycles to failure, right-censored observations, and cyclic load magnitude to be considered within a common framework. This approach allows configurations to be compared over the duration of testing while retaining information from specimens that did not fracture. It is complementary to, rather than a replacement for, conventional fatigue limits and load–life relationships.

Although previous experimental studies have investigated the influence of implant geometry, connection design, and abutment characteristics on fatigue performance, comparisons have generally relied on conventional mechanical descriptors, including fatigue limits and load–life relationships. The specific contribution of the present study is the integration of specimen-level fatigue data, right-censored observations, and cyclic load magnitude within a survival-analysis framework for the comparison of implant–abutment configurations. This approach provides complementary information on configuration-dependent fatigue behaviour beyond that obtained from conventional group-level fatigue descriptors.

Accordingly, the aim of this study was to determine whether fatigue response differed among four titanium implant–abutment configurations using specimen-level data from standardized cyclic loading tests, while accounting for right censoring and cyclic load magnitude. We hypothesized that fatigue response would differ among implant–abutment configurations after adjustment for maximum cyclic load (Pmax).

## 2. Materials and Methods

### 2.1. Implant–Abutment System

The experimental dataset comprised 36 internal-connection implant–abutment assemblies from the IPX system (Galimplant S.L.U., Sarria, Spain). The implant body used in all specimens was the IPX gr IV implant (reference IPX3512), with a diameter of 3.5 mm and a length of 12 mm. The implant body was manufactured from commercially pure Grade 4 titanium and incorporated an internal connection with a hexagonal anti-rotational feature.

The implant surface had been treated by alumina particle blasting followed by acid passivation.

The prosthetic components were manufactured from Grade 5 titanium alloy (Ti6Al4V). These components included the multi-position abutment, the immediate-loading abutment (PTIMUA 4048), and the retaining screw (TMU 4048).

Two multi-position abutment families were evaluated. The MUSLA family comprised slim monoblock multi-position abutments, whereas the MUSA family comprised straight aesthetic anti-rotational 2-piece multi-position abutments.

Four configurations were included: MUSLA 04020 (2 mm transmucosal height), MUSLA 04060 (6 mm transmucosal height), MUSA 04010 (1 mm transmucosal height), and MUSA 04050 (5 mm transmucosal height). Nine specimens were tested for each configuration, resulting in a total of 36 assemblies. The shorter and taller representatives of the corresponding MUSLA and MUSA families were included in the mechanical testing program.

All assemblies were tightened to 30 N·cm using a torque wrench manufactured by Galimplant (Nueva Galimplant S.L.U., Sarria, Spain).

### 2.2. Fatigue Test Configuration

Fatigue testing was conducted in accordance with UNE-EN ISO 14801:2017 [2]., the Spanish adoption of ISO 14801:2016 for dynamic loading tests of endosseous dental implants.

The implants were positioned within copper sleeves and mechanically secured by a jaw system. Compression of the copper sleeve produced uniform fixation around the implant. The test fixture was machined at a nominal angle of 30° relative to the horizontal plane, resulting in a 30° inclination of the implant relative to the loading direction. The documented allowable angular range was 28° to 32°.

The implant position and hemispherical loading member were configured so that the distance between the centre of the loading member and the reference point at the fixation plane was 11 ± 0.5 mm. The fixation plane was positioned 3 mm apical to the nominal bone level.

**Figure 1:**
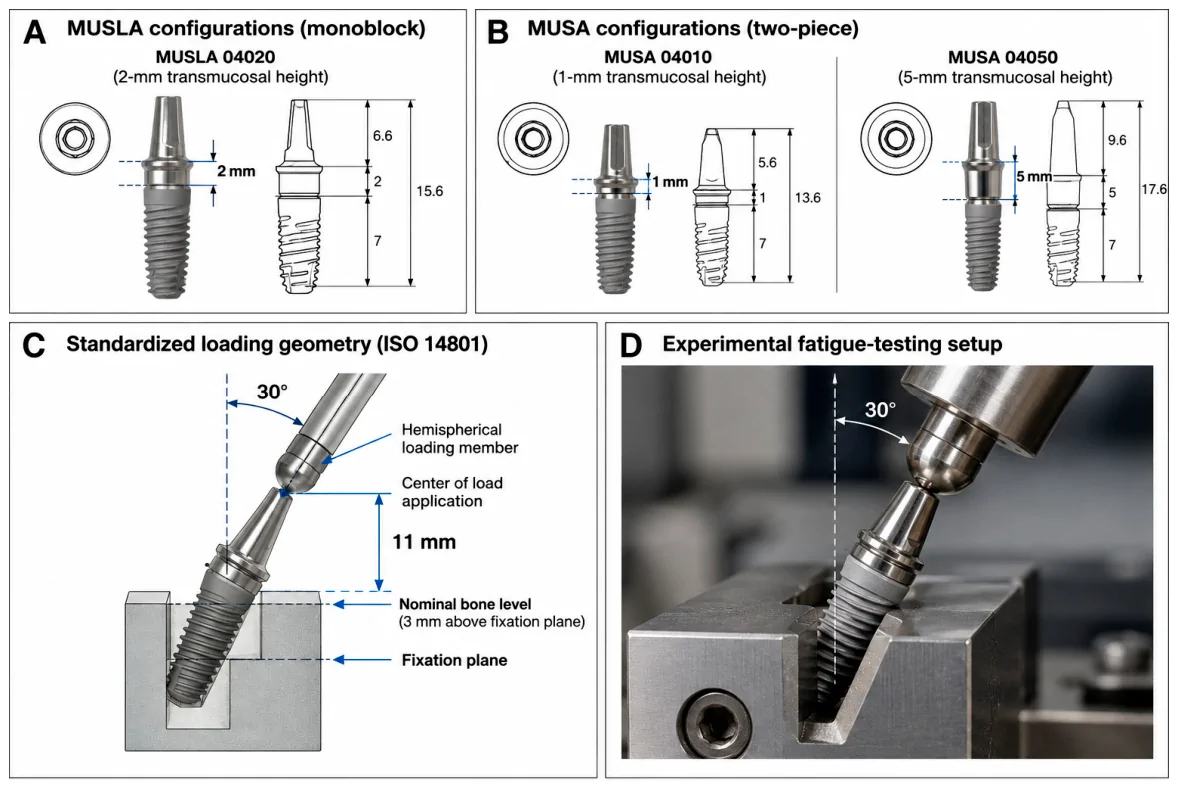
Implant–abutment configurations and experimental fatigue-testing setup. (**A**) MUSLA 04020 (2 mm transmucosal height) and MUSLA 04060 (6 mm transmucosal height) configurations. (**B**) MUSA 04010 (1 mm transmucosal height) and MUSA 04050 (5 mm transmucosal height) configurations. (**C**) Standardized ISO 14801 loading geometry, with the implant axis inclined 30° relative to the loading direction and the centre of load application positioned 11 mm from the fixation plane. The nominal bone level was located 3 mm coronal to the fixation plane. (**D**) Experimental fatigue-testing setup showing the inclined specimen and hemispherical loading member. Different hemispherical loading members were used to compensate for differences in abutment height while maintaining the same loading distance across configurations.

Different hemispherical loading members were used when necessary to compensate for differences in abutment height while maintaining the same 11 mm loading distance. For MUSLA 04020 and MUSLA 04060, the loading members were specifically dimensioned so that the centre of load application remained at the same distance from the fixation plane despite the difference in abutment height. The same principle was applied to MUSA 04010 and MUSA 04050. This geometry standardized the bending lever arm across the tested configurations. The four implant–abutment configurations, standardized loading geometry, and experimental fatigue-testing setup are illustrated in Figure 1.

### 2.3. Cyclic Loading Protocol

Cyclic loading was applied between a maximum load (Pmax) and a minimum load equal to 10% of Pmax (Pmin = 0.1 Pmax), corresponding to a load ratio of R = 0.1. The loading frequency was 15 Hz, and the uncertainty in the applied load was reported to be below 5 N.

The maximum cyclic loads used across the experimental program ranged from 100 to 175 N. The specific load levels differed among configurations and were recorded for each individual specimen.

Testing was performed in air at 20 ± 5 °C and 60% relative humidity.

Fatigue tests were performed using an MTS^®^ 858 MiniBionix mechanical testing system (MTS Systems Corporation, Eden Prairie, MN, USA) equipped with an MTS 661.19F-01 load cell with a capacity of 5-kN. The tests were controlled and monitored using MTS TestSuite^TM^ (version 2025.2) and MultiPurpose TestWare^®^ (MPT, serie 793, version 6.2D) software (MTS Systems Corporation, Eden Prairie, MN, USA).

### 2.4. Experimental Groups and Recorded Outcomes

Nine specimens were analyzed for each of the four implant–abutment configurations, resulting in a total of 36 individual fatigue observations. For each specimen, the recorded variables included maximum cyclic load (Pmax), minimum cyclic load (Pmin), number of completed loading cycles, occurrence or absence of fatigue fracture, and macroscopic failure classification.

The number of specimens reflected the available experimental series, with an equal allocation of nine specimens to each configuration; no formal a priori sample-size calculation was performed. Accordingly, the statistical analyses should be regarded as exploratory, and the estimated effects should be interpreted together with their confidence intervals rather than as definitive estimates of configuration-specific differences.

The load levels for each configuration were established before the fatigue tests included in the present analysis, based on preliminary mechanical testing performed during development of the experimental protocol. These preliminary tests indicated a lower fatigue resistance for the MUSLA 04020 configuration; therefore, lower load levels were included for this configuration to adequately characterize the transition between fracture and run-out. Thus, the load schedules were prespecified for the experimental series analyzed here and were not modified in response to the fatigue outcomes of the specimens included in the present dataset. MUSLA 04020 was tested at maximum loads of 175, 150, 120, and 100 N, whereas MUSLA 04060, MUSA 04010, and MUSA 04050 were tested at maximum loads of 175, 150, and 130 N.

### 2.5. Definition of Fatigue Failure and Right Censoring

The primary outcome was the number of loading cycles completed before fatigue fracture or test termination.

A fatigue event was defined as a specimen in which fatigue fracture was recorded during cyclic loading. Specimens whose tests ended without recorded fracture were treated as right-censored at their recorded termination cycle. Thus, a censored specimen was considered to have remained free of the defined fatigue event up to, but not necessarily beyond, the recorded number of cycles.

Of the 36 specimens, 23 experienced fatigue fracture and 13 ended testing without fracture and were therefore treated as right-censored observations. The original testing records documented the number of completed cycles and fracture status for each specimen but did not consistently document the specific stopping criterion for every non-fractured test. Some specimens completed 5,000,000 cycles without fracture, whereas other non-fractured tests were terminated earlier. The available records did not allow us to determine retrospectively whether 5,000,000 cycles represented a uniformly prespecified run-out threshold for all specimens or to reliably reconstruct the reasons for the earlier termination of other non-fractured tests. Therefore, no common stopping or run-out criterion was imposed retrospectively, and each non-fractured specimen was right-censored at its actual recorded termination cycle.

### 2.6. Failure Classification

The experimental protocol defined L1 as a failure located in the implant body at the level of the fixation plane and D1 as fatigue fracture of the implant body. All specimens with a recorded fatigue failure were classified as L1/D1 in the individual test records. Failure location was therefore considered a descriptive mechanical outcome distinct from fatigue survival.

### 2.7. Statistical Analysis

The individual specimen was the statistical unit. Because some specimens ended testing without fracture, fatigue life was analyzed using a time-to-event framework rather than by conventional comparison of mean cycle counts. The number of completed loading cycles was used as the time variable; fatigue fracture was coded as the event, and specimens ending without fracture were right-censored at the recorded termination cycle.

The primary inferential analysis using a Cox proportional-hazards regression model was specified before examination of the study outcomes [13]. Maximum cyclic load was entered as a continuous covariate and scaled so that hazard ratios represented the effect of a 10 N increase in Pmax. Abutment configuration was included as a categorical predictor, with MUSA 04010 selected a priori as the reference configuration. The model therefore estimated configuration effects on fatigue hazard after adjustment for cyclic load magnitude.

Adjusted hazard ratios (HRs) with 95% confidence intervals (CIs) were calculated. The overall contribution of abutment configuration was assessed by a likelihood-ratio comparison between the full model containing cyclic load and configuration and a reduced model containing cyclic load alone. Pairwise contrasts among configurations were derived from the fitted model, and the Holm procedure was used to control the family-wise error rate across pairwise comparisons [14].

Additional sensitivity analyses, beyond the primary Cox analysis specified before examination of the study outcomes and its model diagnostics, were developed subsequently to assess the robustness of the findings. Given the limited number of fracture events relative to the number of parameters in the primary model, a Cox regression model using Firth’s penalized likelihood correction was additionally fitted as a sensitivity analysis to assess the potential influence of small-sample bias. The penalized model included the same covariates as the primary Cox model, with Pmax scaled per 10 N increase and MUSA 04010 as the reference configuration. Confidence intervals were obtained from the profile penalized likelihood.

The proportional-hazards assumption was assessed using scaled Schoenfeld residuals [15,16] and score tests based on the Kaplan–Meier transformation of time [16,17], together with graphical inspection of residual patterns. Both the global model test and covariate-specific tests were examined. Because the diagnostic identified evidence of time dependence for the MUSLA 04060 term while the global test was not significant, an extended Cox sensitivity model was additionally fitted in counting-process form, allowing the MUSLA 04060 effect to vary with centred Kaplan–Meier-transformed time. Robust standard errors were clustered by specimen in this sensitivity model.

Several additional sensitivity analyses were performed. First, because the original load schedules were not identical among configurations, the Cox analysis was repeated after restriction to the two maximum load levels shared by all four configurations (150 and 175 N). Second, potential nonlinearity of the load effect was assessed by adding a quadratic load term and comparing this model with the corresponding linear-load model by likelihood-ratio testing. Third, a load-by-configuration interaction model was fitted and compared with the additive Cox model to assess whether the association between load magnitude and fatigue hazard differed among configurations.

Influence was assessed by exact leave-one-out analysis. The primary Cox model was refitted 36 times, sequentially excluding one specimen at each iteration, and the resulting configuration-effect estimates were examined for changes in direction and magnitude.

Analyses were performed using Stata 19 (StataCorp LLC, College Station, TX, USA). All tests were 2-sided, with statistical significance defined as *p* < 0.05. Given the limited number of specimens per configuration, effect estimates were interpreted together with their 95% CIs and the results of the sensitivity and influence analyses.

### 2.8. Generative Artificial Intelligence (GenAI)

Generative artificial intelligence (GenAI) was used solely as an ancillary language-editing tool during manuscript preparation, mainly for minor wording, clarity and readability improvements. All AI assisted text was reviewed and approved by the authors, who take full responsibility for the final manuscript.

## 3. Results

### 3.1. Observed Fatigue Outcomes

Thirty-six implant–abutment assemblies were included in the analysis, with nine specimens in each configuration. Twenty-three specimens sustained a recorded fatigue fracture, whereas 13 tests ended without fracture and were treated as right-censored at the recorded termination cycle. All recorded fatigue fractures were classified as L1/D1, corresponding to fatigue fracture of the implant body at the fixation plane.

**Figure 2:**
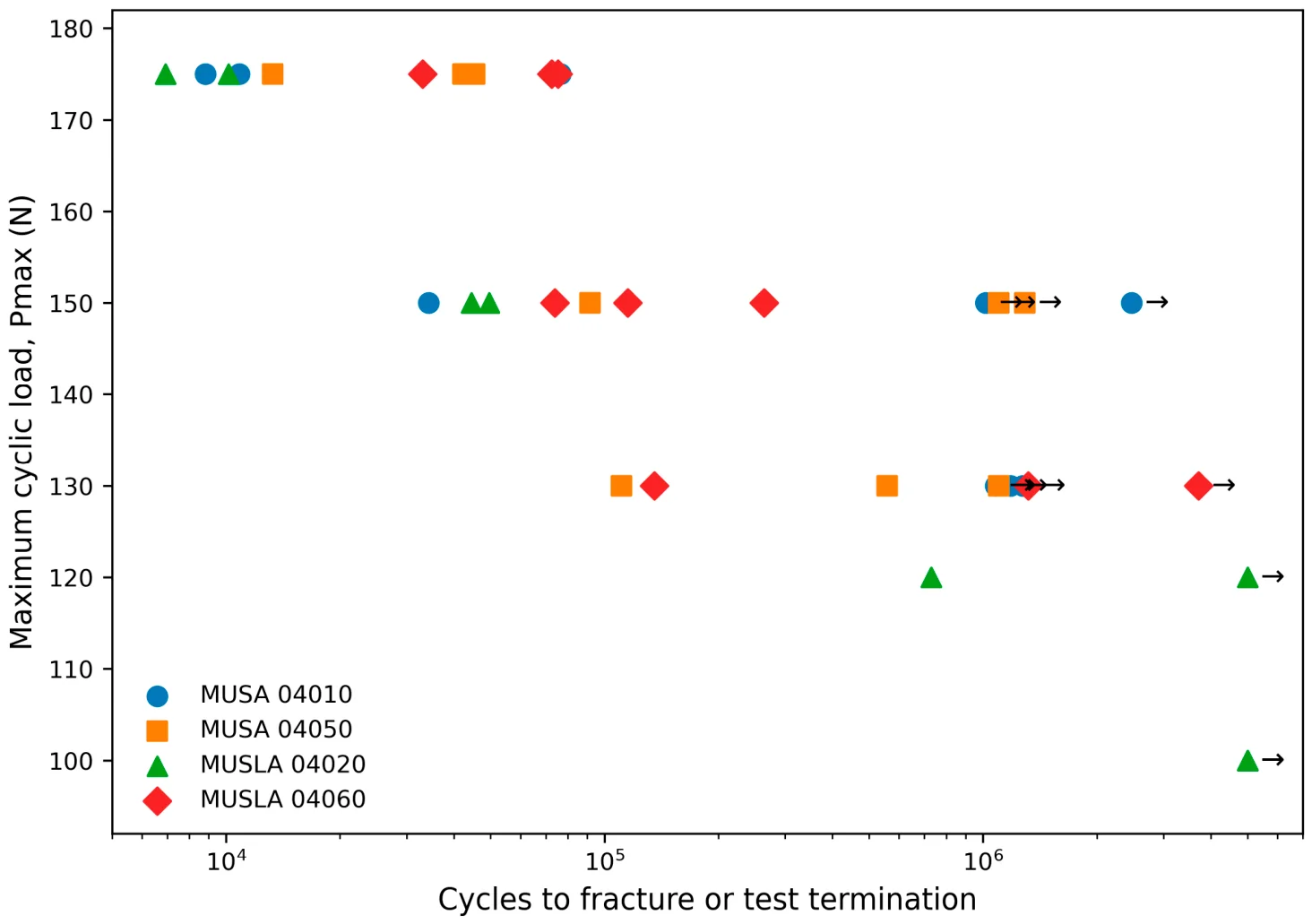
Observed load–life relationship for the 36 implant–abutment assemblies. Each symbol represents one specimen. Arrows identify tests that ended without recorded fracture and were treated as right-censored at the corresponding cycle count.

At a maximum cyclic load of 175 N, all tested specimens fractured in all four configurations. At 150 N, all MUSLA 04020 (2/2) and MUSLA 04060 (3/3) specimens fractured, whereas one of three specimens fractured in each MUSA configuration. At 130 N, fracture occurred in one of three MUSLA 04060 specimens, one of three MUSA 04010 specimens, and two of three MUSA 04050 specimens. For MUSLA 04020, one of two specimens fractured at 120 N and none of the three specimens fractured at 100 N (Table 1; Figure 2).

### 3.2. Primary Load-Adjusted Cox Analysis

Maximum cyclic load was strongly associated with fatigue hazard. In the primary Cox model, each 10 N increase in Pmax was associated with a hazard ratio (HR) of 2.52 (95% CI, 1.75–3.62; *p* < 0.001). After adjustment for cyclic load, the overall contribution of abutment configuration was statistically significant (likelihood-ratio chi-square = 9.17, df = 3, *p* = 0.027).

**Figure 3:**
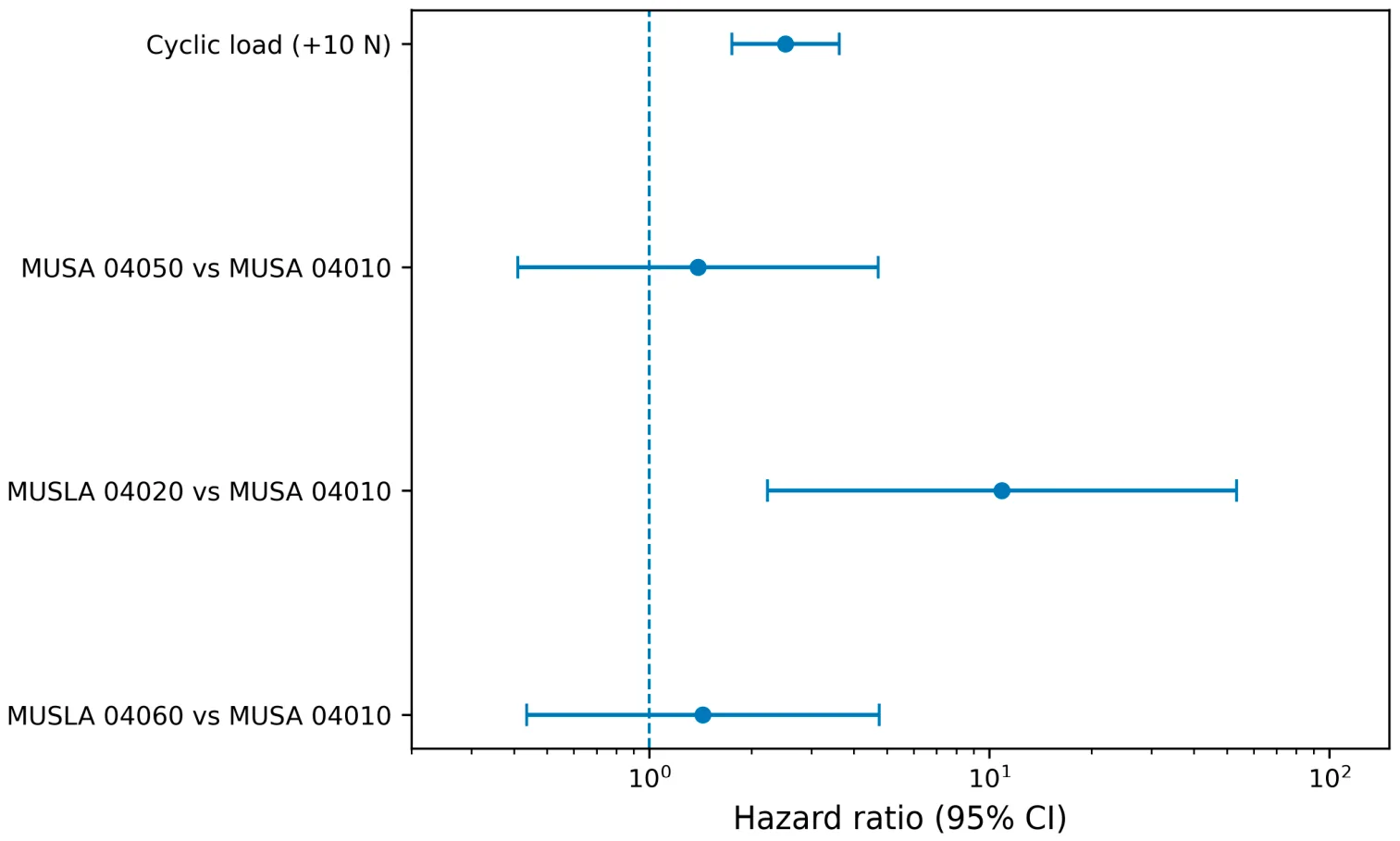
Adjusted hazard ratios and 95% confidence intervals from the primary Cox proportional-hazards model. Configuration estimates are relative to MUSA 04010; the cyclic-load estimate corresponds to a 10 N increase in Pmax. The vertical dashed line indicates HR = 1.

With MUSA 04010 as the reference configuration, MUSLA 04020 was associated with a higher fatigue hazard (HR = 10.89; 95% CI, 2.23–53.33; *p* = 0.003). MUSA 04050 (HR = 1.39; 95% CI, 0.41–4.71; *p* = 0.596) and MUSLA 04060 (HR = 1.44; 95% CI, 0.44–4.75; *p* = 0.551) did not differ significantly from MUSA 04010 in the primary model (Table 2; Figure 3).

### 3.3. Multiplicity-Adjusted Pairwise Comparisons

After Holm adjustment for the six pairwise configuration comparisons, MUSLA 04020 remained significantly different from MUSA 04010 (adjusted *p* = 0.019), MUSA 04050 (adjusted *p* = 0.029), and MUSLA 04060 (adjusted *p* = 0.029). The estimated fatigue hazard for MUSLA 04020 was 10.89 times that of MUSA 04010, 7.84 times that of MUSA 04050, and 7.58 times that of MUSLA 04060. None of the pairwise comparisons among MUSA 04010, MUSA 04050, and MUSLA 04060 were significant after multiplicity adjustment (Table 3).

### 3.4. Proportional-Hazards Diagnostics

The global scaled-Schoenfeld-residual score test did not provide evidence of a global departure from proportional hazards (chi-square = 7.67, df = 4, *p* = 0.105). Covariate-specific tests were non-significant for cyclic load (*p* = 0.241), MUSA 04050 (*p* = 0.896), and MUSLA 04020 (*p* = 0.871). A covariate-specific signal of time dependence was detected for MUSLA 04060 (chi-square = 4.05, *p* = 0.044).

**Figure 4:**
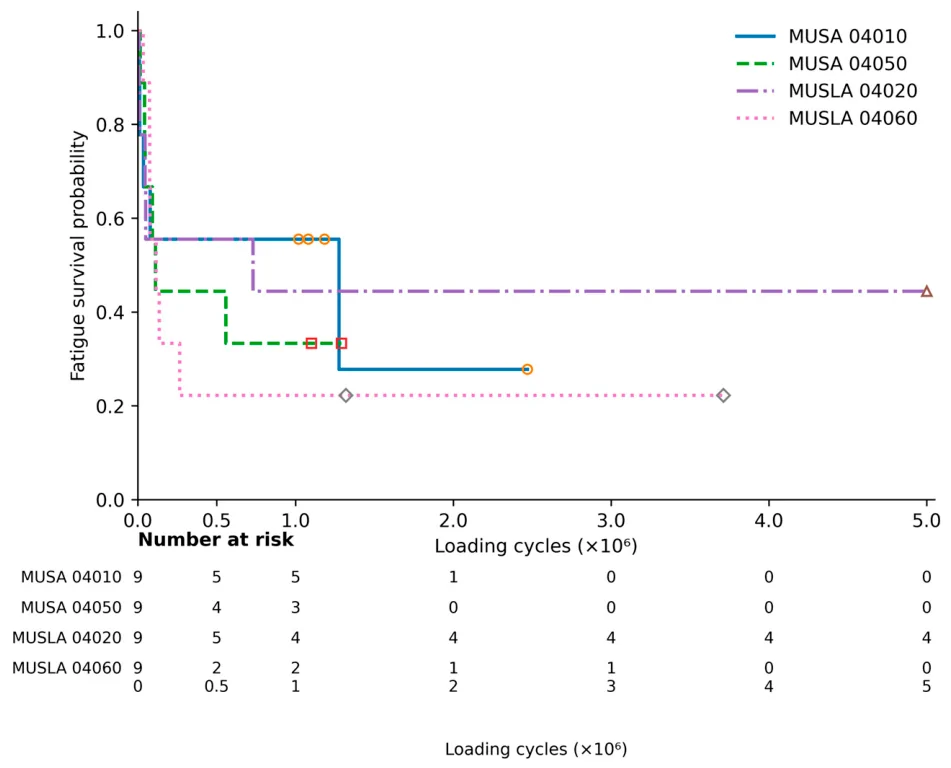
Kaplan–Meier estimates of fatigue survival stratified by implant–abutment configuration. Censored observations are indicated by censoring marks, and the numbers at risk are shown below the plot.

Kaplan–Meier curves stratified by implant–abutment configuration are shown in Figure 4, with censored observations indicated and numbers at risk reported below the plot. The curves provide a descriptive representation of the observed fatigue survival patterns across configurations and complement the model-based assessment of the proportional-hazards assumption.

Accordingly, an extended Cox sensitivity model allowing the MUSLA 04060 coefficient to vary with centred Kaplan–Meier-transformed time was fitted. In this model, the higher fatigue hazard for MUSLA 04020 relative to MUSA 04010 was retained (HR = 12.46; 95% CI, 2.07–75.01; *p* = 0.006), despite allowing the MUSLA 04060 effect to vary with time. The time-dependent MUSLA 04060 term had *p* = 0.048.

### 3.5. Sensitivity and Influence Analyses

A Firth-penalized Cox regression was additionally performed to assess the potential influence of the limited number of fracture events relative to the number of model parameters. The results were consistent with those of the primary Cox model. The HR for each 10 N increase in Pmax was 2.42 (95% CI, 1.76–3.57; *p* < 0.001). Relative to MUSA 04010, the HRs were 1.35 (95% CI, 0.42–4.56; *p* = 0.612) for MUSA 04050, 9.96 (95% CI, 2.25–49.10; *p* = 0.003) for MUSLA 04020, and 1.38 (95% CI, 0.44–4.62; *p* = 0.576) for MUSLA 04060. Thus, the Firth-penalized analysis retained the higher fatigue hazard for MUSLA 04020 relative to MUSA 04010, while MUSA 04050 and MUSLA 04060 remained non-significantly different from the reference configuration.

Restriction to the load levels common to all four configurations (150 and 175 N) retained 22 specimens and 18 fracture events. The overall configuration effect remained statistically significant (likelihood-ratio chi-square = 9.82, df = 3, *p* = 0.020). In this restricted analysis, MUSLA 04020 remained associated with a higher fatigue hazard than MUSA 04010 (HR = 16.92; 95% CI, 2.45–116.67; *p* = 0.004).

**Figure 5:**
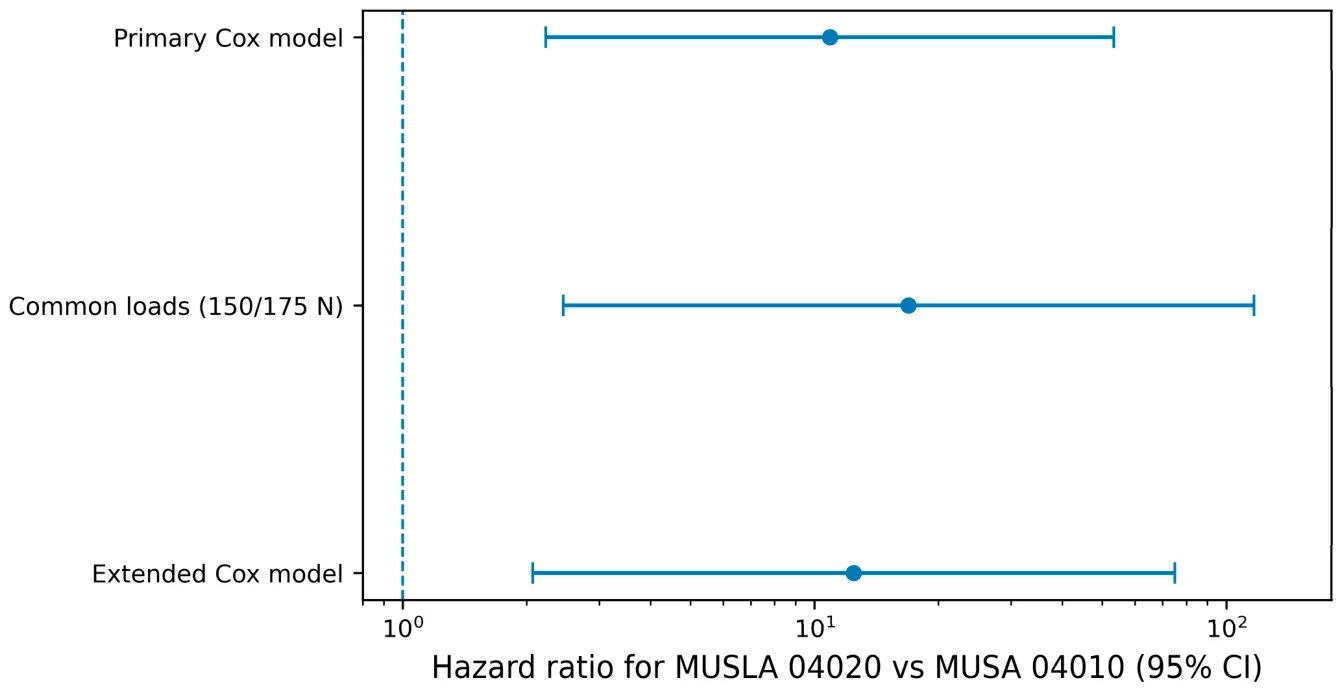
Robustness of the MUSLA 04020 versus MUSA 04010 hazard-ratio estimate across the primary Cox model, the analysis restricted to common load levels (150 and 175 N), and the extended Cox sensitivity model allowing time dependence for MUSLA 04060. Points are hazard ratios and bars are 95% confidence intervals.

Adding load-by-configuration interaction terms did not improve model fit (likelihood-ratio chi-square = 2.25, df = 3, *p* = 0.523), providing no evidence that the load effect differed among the four configurations. Addition of a quadratic load term also did not improve model fit (likelihood-ratio chi-square = 0.001, df = 1, *p* = 0.971) (Table 4, Figure 5).

In the leave-one-out analysis, the estimated HR for MUSLA 04020 versus MUSA 04010 remained above 1 in all 36 refitted models and ranged from 7.31 to 17.43. Thus, the direction of the primary association was not dependent on any single specimen.

## 4. Discussion

Fatigue performance of implant-supported assemblies is influenced by both loading conditions and the structural characteristics of the implant–abutment assembly. Within this context, the present study aimed to determine whether fatigue response differed among four titanium implant–abutment configurations after accounting for right censoring and cyclic load magnitude. The main finding was that fatigue hazard was associated with both maximum cyclic load and abutment configuration. For every 10 N increase in Pmax, the estimated hazard increased 2.52-fold. After adjustment for load, configuration remained significant, with MUSLA 04020 showing a higher hazard than MUSA 04010, MUSA 04050, and MUSLA 04060. No significant differences were found among the latter three configurations after multiplicity correction.

### 4.1. Principal Findings

This agrees with previous experimental studies showing that fatigue behaviour can vary with structural and connection-related features as well as applied load [3,4,5,10]. It is also important for interpreting these results because the four groups were not simply different transmucosal heights within one abutment design.

The association involving MUSLA 04020 remained in the same direction across the different analytical approaches. It was retained after restriction to the common 150- and 175 N load levels, in the Firth-penalized Cox model, in all 36 leave-one-out analyses, and in the extended Cox model accounting for the time-dependent behaviour detected for MUSLA 04060. However, the magnitude of the estimated association varied across models.

Therefore, the individual hazard-ratio estimates should not be interpreted as precise measures of effect size. In particular, the wide confidence interval around the estimate for MUSLA 04020 versus MUSA 04010 indicates substantial uncertainty regarding the magnitude of this association. The more robust finding is the consistent direction of the association across the different analytical approaches.

The magnitude of the estimated effects should nevertheless be viewed in light of the sample size. Only nine specimens were available for each configuration, and several confidence intervals were consequently wide. Although the sensitivity analyses support the consistency of the main association across different analytical approaches, they cannot overcome the uncertainty inherent in a small dataset. Differences in fatigue response between implant–abutment configurations have also been reported in previous experimental studies [11,18,19,20,21].

All 23 recorded fractures were classified as L1/D1, corresponding to fracture of the implant body at the fixation plane. Thus, the configurations differed in fatigue survival despite having the same macroscopic fracture classification. This distinction matters because the final fracture location does not indicate how long the assembly resisted cyclic loading.

Similar findings have been reported in experimental and numerical–experimental studies, where different fatigue resistance or predicted lifetime was observed despite comparable terminal fracture regions [5,22,23,24,25].

In this study, fatigue survival and macroscopic failure classification provide complementary information on mechanical performance and should not be considered interchangeable outcomes.

### 4.2. Biomechanical Interpretation of Configuration-Dependent Fatigue Behaviour

The configuration effect should be considered at the level of the complete implant–abutment assembly. During cyclic loading, the implant, abutment, retaining screw, connection and loading geometry act as a coupled mechanical system. Fatigue response can consequently reflect differences in geometry, material properties, preload and load transfer acting together [3,26,27,28]. Previous experimental and computational studies have shown similar effects of structural design on fatigue resistance within individual implant systems [23,27,28].

The experiment does not show which mechanical feature produced the higher hazard observed for MUSLA 04020. Local stress distributions and crack-initiation sites were not investigated, so the available data cannot distinguish between the possible geometric or mechanical explanations.

The results also do not support interpretation based on transmucosal height alone. The 2 mm and 6 mm MUSLA configurations behaved differently after adjustment for load, whereas the 1 mm and 5 mm MUSA configurations did not show a significant difference. Within these data, neither a shorter nor a taller abutment produced a consistent effect across the two families.

Previous work has shown that the influence of abutment or restoration height and other geometric variables can depend on the implant system being studied [7,8,9,29]. The differences observed here should therefore be considered specific to the configurations tested, rather than evidence for a general effect of transmucosal height.

An additional structural difference between the tested families is that MUSLA abutments have a monoblock configuration, whereas MUSA abutments have a two-piece construction. This distinction may influence load transfer and mechanical behaviour and should therefore be considered when interpreting the configuration effect. However, both MUSLA 04020 and MUSLA 04060 share the monoblock design, while only MUSLA 04020 showed a significantly higher fatigue hazard. Thus, monoblock versus two-piece construction alone cannot account for the observed pattern, further supporting interpretation of the fatigue differences at the level of the complete implant–abutment assembly rather than attribution to a single design feature.

Screw-joint mechanics represent another potential contributor to the fatigue response of the assemblies. All abutment screws were tightened to the same nominal torque in the present study; however, the resulting preload was not directly measured, and the effects of repeated tightening or retightening were not evaluated. Previous experimental evidence has shown that repeated tightening can influence screw stability and preload-related behaviour. Therefore, although tightening torque was standardized across configurations, possible differences in screw-joint behaviour cannot be excluded as part of the overall mechanical response of the tested assemblies [30].

The loading arrangement is another relevant consideration. Different hemispherical loading members were used to compensate for the different abutment heights while keeping the distance between the centre of load application and the fixation plane at 11 mm in all configurations.

This makes a simple explanation based on a different external bending lever arm less likely. At the same time, height was not varied independently of the other geometric features defining the MUSLA and MUSA assemblies. Its individual contribution therefore cannot be separated from those other features. Connection dimensions, platform geometry and related design variables have also been associated with differences in fatigue behaviour in previous studies [4,31,32,33].

The common L1/D1 classification provides another example of why the mechanical response cannot be reduced to a single outcome. The experiment does not demonstrate different macroscopic fracture mechanisms, nor can it establish whether crack initiation occurred at the same microscopic site in every group. Assemblies with similar terminal fracture regions can nevertheless have substantially different fatigue lives.

Experimental and computational studies have reported comparable situations, with changes in fatigue resistance occurring without an obvious change in the final fracture region [23,26,28].

Finite element analysis and fractographic examination would be needed to identify the mechanical basis of the observed configuration effect.

The relationship with Pmax is also compatible with the expected dependence of cyclic fatigue on applied load. Within the tested range, higher Pmax was associated with a higher fracture hazard. Adding a quadratic load term or a load-by-configuration interaction did not improve model fit. The additive load-adjusted model consequently provides a reasonable description of these data, although the analyses do not establish strict linearity or identical load sensitivity across configurations. Loading angle, direction and multiaxial stress state may also influence implant fatigue measurements [34,35,36].

### 4.3. Relationship with Previous Experimental and Computational Evidence

The results are broadly consistent with experimental evidence showing that implant–abutment connection architecture can influence fatigue behaviour. Gil et al. reported design-dependent differences in fatigue and fracture behaviour [4]. Choi et al. likewise found that changes in internal-connection design affected breaking load and fatigue limit without producing a significant difference in fracture-area distribution [5].

Nicolas-Silvente et al. reported differences in fatigue load limits among implant systems with different connection designs and platform diameters [22]. Other comparisons of internal tapered and ferrulized connections have also shown system-dependent differences in cyclic mechanical behaviour [18,19]. These studies support an influence of assembly configuration on fatigue behaviour, but they do not identify a universally superior connection design.

Studies using the ISO 14801 protocol have reached similar conclusions. Thomé et al. compared different implant–abutment connections under standardized dynamic loading [20], while On et al. reported differences among systems with different internal-connection designs [21].

Comparison across these studies has an important limitation: several structural variables may change at the same time, making it difficult to attribute a fatigue difference to one feature. The same issue applies here because MUSLA and MUSA represent complete configurations rather than experimental manipulations of a single geometric parameter. The present results should not be interpreted as a general ranking of abutment families or connection types.

Implant diameter is another variable that can affect fatigue performance [6,10,36,37,38,39,40]. Changes in diameter and effective cross-section alter the mechanical response, but findings obtained with other implant dimensions cannot be transferred directly to the 3.5 mm implant evaluated in this study.

Computational work offers several possible explanations for configuration-dependent fatigue behaviour. Differences in cone angle, connection dimensions, preload and prosthetic-platform geometry have been associated with changes in calculated stress or fatigue performance in other systems [26,28,31,32,33,41]. None of these mechanisms can be assigned to the present MUSLA–MUSA difference because the tested assemblies were not examined by finite element analysis.

Retaining specimen-level fatigue times and right-censored observations provides additional information beyond conventional fatigue measures. Conventional fatigue limits, fracture loads and load–life relationships remain important engineering descriptors [10,11,12,42], but they do not use all of the information available when specimens are tested at different loads and some tests end without fracture. Survival analysis therefore adds another description of the experimental results rather than replacing the conventional measures.

### 4.4. Methodological Implications, Strengths, and Limitations

The time-to-event approach allowed the 13 non-fractured specimens to remain in the analysis as right-censored observations at their recorded termination cycles. These specimens show that the defined fatigue event had not occurred when testing stopped, although their eventual fatigue life is unknown. This avoids treating run-outs as failures and retains the survival information available up to termination [1,11,12].

Adjustment for Pmax was important because the load schedules were not identical across configurations. MUSLA 04020 included tests at 100 and 120 N, whereas the other configurations had 130 N as their lowest load level. The Cox model accounted for this difference, and the separate analysis at the common 150 and 175 N levels provided a more direct comparison under the same loads. Survival methods have also been used previously in implant fatigue research [8,43].

At the specimen level, the analysis incorporated fatigue time, censoring, cyclic load and configuration. Multiple comparisons, model diagnostics, sensitivity analyses and leave-one-out assessment were used to examine whether the principal association was sensitive to the analytical choices or to individual observations.

The experimental conditions were otherwise kept as constant as possible. Implant body and dimensions, component materials, tightening torque, loading angle and nominal loading distance were controlled across the four groups.

Testing followed the ISO 14801 framework, providing standardized conditions for comparative dynamic mechanical evaluation [2]. The same number of specimens was tested in each configuration, and the main association remained consistent across the Firth-penalized, common-load, time-dependent, and leave-one-out sensitivity analyses. These strengths should nevertheless be considered within the limits of a laboratory experiment; the results describe the behaviour of the tested assemblies and do not establish clinical performance.

The small sample size and limited number of fracture events relative to the number of parameters in the Cox model are important limitations. No formal a priori sample-size calculation was performed because the study was based on the available experimental series of nine specimens per configuration. Accordingly, statistical analyses should be considered exploratory. With 23 fracture events and four model parameters, the events-per-parameter ratio was low, which may reduce the stability and precision of the coefficient estimates and is consistent with the wide confidence intervals observed for some configuration effects. The Firth-penalized Cox sensitivity analysis yielded closely similar results and retained the association observed for MUSLA 04020, reducing concern regarding small-sample coefficient bias. Nevertheless, the wide confidence intervals remained, and the magnitude of individual hazard-ratio estimates should therefore be interpreted cautiously. The unequal load schedules are a further limitation. Although the common-load analysis retained the main association, that analysis included only 22 specimens and 18 fracture events. Another issue concerns the non-fractured specimens. The original experimental records documented the number of completed cycles and fracture status but did not consistently document the specific reason for termination of every non-fractured test. Some specimens completed 5,000,000 cycles without fracture, whereas other non-fractured tests were terminated earlier. The available records did not allow us to determine retrospectively whether 5,000,000 cycles represented a uniformly prespecified run-out threshold for all specimens or to reliably reconstruct the reasons for the earlier termination of other non-fractured tests. Each specimen was therefore censored at its actual recorded termination cycle. Although this preserves the available survival information up to termination, the assumption of non-informative censoring cannot be fully verified and should be considered when interpreting the Cox model estimates.

A more fundamental limitation concerns the relationship between abutment family and transmucosal height. The two variables were not independently manipulated in a factorial design; each height was part of a particular MUSLA or MUSA configuration. As a result, the observed effect cannot be separated into independent contributions from height, abutment family and the other geometric features of the assemblies. In particular, the higher hazard of MUSLA 04020 should not be interpreted as a causal effect of its 2 mm transmucosal height.

The proportional-hazards diagnostics identified a covariate-specific time-dependence signal for MUSLA 04060, although the global test was not significant. Allowing this effect to vary with time did not materially change the main MUSLA 04020 association. Even so, the number of fracture events limits the complexity of models that can be supported. Similarly, the absence of a statistically detectable load-by-configuration interaction or load nonlinearity means only that such effects were not demonstrated in this dataset; it does not show that they are absent. Maximum applied load also does not capture all multiaxial or notch-related aspects of implant fatigue [34].

Finally, the laboratory conditions limit external validity. ISO 14801 is designed for standardized comparison rather than direct simulation of mastication or prediction of clinical service life [2]. The present tests used unidirectional cyclic loading in air at 15 Hz, whereas loading direction, angle and environmental conditions can influence titanium fatigue behaviour [1,34,35,36,44,45,46]. The hazard ratios should be interpreted as comparative estimates for this experimental system, not as estimates of clinical failure risk or service life.

### 4.5. Future Perspectives

Future work could combine specimen-level survival analysis with a more balanced experimental design, larger sample sizes and predefined, uniform censoring thresholds. Conventional F-N or S-N relationships and fatigue-limit estimates should remain part of that analysis because they provide engineering information that complements survival-based methods [10,11,12]. A larger and more balanced dataset would also permit a more informative assessment of possible nonlinear load effects and interactions between load and configuration.

The mechanical basis of the configuration effect remains unresolved. Finite element modelling could compare local stress distributions in the MUSLA and MUSA assemblies, while fractographic examination could provide information on crack initiation and propagation. Similar approaches have been used to relate structural configuration to fatigue-sensitive regions in other implant systems [23,25,28,30]. Such analyses would be needed before attributing the observed survival difference to a particular stress distribution or crack-initiation mechanism.

The configurations should also be tested under a broader range of mechanical and environmental conditions. Multidirectional or multiaxial loading, together with aqueous or oral-like environments, would address factors not represented by the present dry-air, unidirectional protocol [34,35,46]. ISO 14801 remains useful for standardized comparisons, but results obtained under this protocol should not be treated as direct predictions of clinical longevity.

## 5. Conclusions

Within the standardized laboratory conditions of this study, fatigue survival in the tested titanium implant–abutment assemblies was associated with maximum cyclic load and abutment configuration. Each 10 N increase in Pmax was associated with a 2.52-fold higher fatigue hazard, and configuration remained significant after adjustment for load. MUSLA 04020 showed a higher estimated fatigue hazard than MUSA 04010, MUSA 04050, and MUSLA 04060; however, the wide confidence intervals indicate substantial uncertainty regarding the magnitude of these configuration effects. The association involving MUSLA 04020 remained in the same direction across the Firth-penalized, common-load, time-dependent, and leave-one-out analyses, although the magnitude of the estimated effect varied across models. Accordingly, greater emphasis should be placed on the consistency and direction of the observed association than on the precise magnitude of the individual hazard-ratio estimates.

The observed differences apply to the complete implant–abutment configurations. Transmucosal height varied with abutment family and other geometric features and was not independently manipulated; its contribution therefore cannot be separated from the other configuration-related effects. Despite differences in fatigue survival, all recorded fractures had the same L1/D1 macroscopic classification.

Specimen-level survival analysis allowed fractured and right-censored observations to be analyzed together while accounting for cyclic load and configuration. This provides information complementary to conventional fatigue limits and load–life relationships. Given the experimental and exploratory nature of the study, the limited sample size, and the uncertainty associated with the non-uniform termination of some non-fractured tests, these findings should be considered preliminary and require confirmation in larger, prospectively standardized experimental studies. The reported hazard ratios describe relative fatigue hazard under the experimental test conditions and should not be interpreted as estimates of clinical failure risk or service life, nor should the observed differences among configurations be used to guide clinical abutment selection directly.

## Figures and Tables

**Table 1:** Observed fatigue outcomes by implant–abutment configuration and maximum cyclic load.

Group	Height (mm)	Pmax (N)	n	Failures	Run-Outs	Min Cycles	Max Cycles
MUSA 04010	1	175	3	3	0	8825	76,466
MUSA 04010	1	150	3	1	2	34,270	2,468,434
MUSA 04010	1	130	3	1	2	1,080,089	1,274,476
MUSA 04050	5	175	3	3	0	13,286	45,335
MUSA 04050	5	150	3	1	2	91,534	1,290,000
MUSA 04050	5	130	3	2	1	110,845	1,100,000
MUSLA 04020	2	175	2	2	0	6920	10,148
MUSLA 04020	2	150	2	2	0	44,488	49,605
MUSLA 04020	2	120	2	1	1	729,426	5,000,000
MUSLA 04020	2	100	3	0	3	5,000,000	5,000,000
MUSLA 04060	6	175	3	3	0	33,074	75,415
MUSLA 04060	6	150	3	3	0	73,879	264,154
MUSLA 04060	6	130	3	1	2	135,555	3,710,762

Note: Cycle ranges are observed values and include both fracture times and test-termination times for right-censored specimens.

**Table 2:** Primary Cox proportional-hazards model for fatigue fracture.

Predictor	Adjusted HR	95% CI Lower	95% CI Upper	*p*-Value
Cyclic load (+10 N)	2.52	1.75	3.62	<0.001
MUSA 04050	1.39	0.41	4.71	0.596
MUSLA 04020	10.89	2.23	53.33	0.003
MUSLA 04060	1.44	0.44	4.75	0.551

Note: MUSA 04010 was the reference configuration. Global configuration effect: likelihood-ratio chi-square = 9.17; df = 3; *p* = 0.027.

**Table 3:** Pairwise configuration contrasts from the primary Cox model with Holm adjustment.

Comparison	HR	95% CI Lower	95% CI Upper	Unadjusted *p*	Holm-Adjusted *p*
MUSA 04050 vs. MUSA 04010	1.39	0.41	4.71	0.596	1.000
MUSLA 04020 vs. MUSA 04010	10.89	2.23	53.33	0.003	0.019
MUSLA 04060 vs. MUSA 04010	1.44	0.44	4.75	0.551	1.000
MUSLA 04020 vs. MUSA 04050	7.84	1.82	33.77	0.006	0.029
MUSLA 04060 vs. MUSA 04050	1.03	0.33	3.24	0.954	1.000
MUSLA 04020 vs. MUSLA 04060	7.58	1.72	33.34	0.007	0.029

**Table 4:** Model diagnostics and sensitivity analyses.

Analysis	Estimate/Statistic	df	*p*-Value	Interpretation
Global proportional-hazards test	chi-square = 7.67	4	0.105	No global evidence of non-proportionality
MUSLA 04060 PH diagnostic	chi-square = 4.05	1	0.044	Covariate-specific time-dependence signal
Extended Cox: MUSLA 04020 vs. MUSA 04010	HR = 12.46 (95% CI 2.07–75.01)	-	0.006	Primary association retained
Firth-penalized Cox: Pmax, per 10 N increase	HR = 2.42 (95% CI 1.76–3.57)	-	<0.001	Load effect retained
Firth-penalized Cox: MUSA 04050 vs. MUSA 04010	HR = 1.35 (95% CI 0.42–4.56)	-	0.612	No significant difference
Firth-penalized Cox: MUSLA 04020 vs. MUSA 04010	HR = 9.96 (95% CI 2.25–49.10)	-	0.003	Primary association retained
Firth-penalized Cox: MUSLA 04060 vs. MUSA 04010	HR = 1.38 (95% CI 0.44–4.62)	-	0.576	No significant difference
Common-load analysis: global configuration	chi-square = 9.82	3	0.020	Configuration effect retained
Common-load analysis: MUSLA 04020 vs. MUSA 04010	HR = 16.92 (95% CI 2.45–116.67)	-	0.004	Association retained
Load x configuration interaction	chi-square = 2.25	3	0.523	No evidence of interaction
Quadratic load term	chi-square = 0.001	1	0.971	No evidence supporting nonlinearity
Leave-one-out: MUSLA 04020 HR range	7.31–17.43	-	-	Direction retained in all 36 refits

## Data Availability

The original data presented in this study are openly available in Zenodo at https://doi.org/10.5281/zenodo.22306621.
